# Rac-deficient cerebellar granule neurons die before they migrate to the internal granule layer

**DOI:** 10.1038/s41598-022-19252-y

**Published:** 2022-09-01

**Authors:** Kei-ichi Katayama, Yi Zheng, Norimitsu Inoue

**Affiliations:** 1 Department of Molecular Genetics, Wakayama Medical University, 811-1 Kimiidera, Wakayama, 641-8509 Japan; 2 Division of Experimental Hematology and Cancer Biology, Cincinnati Children’s Hospital Medical Center, Cincinnati, OH 45229 USA

**Keywords:** Neuroscience, Cell death in the nervous system, Cellular neuroscience, Development of the nervous system

## Abstract

Granule neurons are the most common cell type in the cerebellum. They are generated in the external granule layer and migrate inwardly, forming the internal granule layer. Small Rho GTPases play various roles during development of the nervous system and may be involved in generation, differentiation and migration of granule neurons. We deleted *Rac1*, a member of small Rho GTPases, by GFAP-Cre driver in cerebellar granule neurons and Bergmann glial cells. *Rac1*^*flox/flox*^; *Cre* mice showed impaired migration and slight reduction in the number of granule neurons in the internal granule layer. Deletion of both *Rac1* and *Rac3* resulted in almost complete absence of granule neurons. Rac-deficient granule neurons differentiated into p27 and NeuN-expressing post mitotic neurons, but died before migration to the internal granule layer. Loss of *Rac3* has little effect on granule neuron development. *Rac1*^*flox/flox*^; *Rac3*^+/−^; *Cre* mice showed intermediate phenotype between *Rac1*^*flox/flox*^; *Cre* and *Rac1*^*flox/flox*^; *Rac3*^−/−^; *Cre* mice in both survival and migration of granule neurons. *Rac3* itself seems to be unimportant in the development of the cerebellum, but has some roles in *Rac1*-deleted granule neurons. Conversely, overall morphology of *Rac1*^+*/flox*^; *Rac3*^−/−^; *Cre* cerebella was normal. One allele of *Rac1* is therefore thought to be sufficient to promote development of cerebellar granule neurons.

## Introduction

Small Rho GTPases play a variety of roles during development of the nervous system, including neurogenesis, differentiation, migration, dendritogenesis, axon guidance and synapse formation^1,2^. Rac subfamily of small Rho GTPases consists of three members: Rac1, Rac2 and Rac3. Physiological functions of Rac1 have been studied using knockout mice. Although Rac2 expression can be found only in hematopoietic cells^3^, Rac3 is ubiquitously expressed in the nervous system^4,5^. Rac3 may therefore have some roles in Rac1-deficient neuronal cells, creating difficulty in studying the functions of “Rac” in the nervous system.

Granule neurons are the most common cell type in the cerebellum. Cerebellar granule neuron precursors are generated in the rhombic lip and migrate tangentially along the surface of the cerebellar primordium to form the external granular layer (EGL). Granule neuron precursors continue to proliferate in the EGL and then exit the cell cycle to differentiate into granule neurons. Differentiated granule neurons then migrate inwardly past the Purkinje cells and eventually form the internal granular layer (IGL), where they extend their axons into the molecular layer to form parallel fibers^6^. Cerebellar granule neurons were thought to be devoid of *Rac3* and predominantly express *Rac1*^4^, and deletion of *Rac1* in cerebellar granule neurons resulted in impaired migration and axon formation^7^. Detailed analysis revealed, however, that cerebellar granule neurons express not only *Rac1,* but also *Rac3* during development, and that deletion of *Rac3* in addition to *Rac1* resulted in much more severe phenotype than that of *Rac1*-knockout mice^8^. Deletion of *Rac1* by Atoh1-Cre driver in addition to *Rac3* resulted in agenesis of the IGL in the anterior medial part of the cerebellum. Cerebellar granule neurons deficient in both *Rac1* and *Rac3* can differentiate normally until the expression of NeuN, a marker for postmitotic cerebellar granule neurons. After that, however, they exhibit defects in neuritogenesis and die by apoptosis in the deep layer of the EGL before migrating to the IGL, resulting in agenesis of the IGL.

Small Rho GTPases play a variety of roles during development and they may play different roles within the different developmental stages. For example, deletion of *Rhoa* in the cerebral cortical neurons by FoxG1-Cre driver induced disruption of adherens junctions and hyperproliferation of neural progenitor cells^9^, whereas that by Emx1-Cre mouse resulted in double cortex formation due to radial glial scaffold disruption^10^. To elucidate the variety of roles of small Rho GTPases, each of them need to be deleted by different Cre drivers that can induce Cre/loxP recombination within different developmental stages. In the present study, we therefore deleted *Rac1* in cerebellar granule neurons and Bergmann glial cells using GFAP-Cre mice in addition to *Rac3* and examined the roles of Rac in the development of the cerebellum.

## Materials and methods

### Animals

The following mouse strains were used in this study: *Rac1*-floxed^11,12^, *Rac3*-knockout^5^ and GFAP-Cre^13^. Mice of both sexes were used. To obtain *Rac1*^+*/flox*^; *Cre* and *Rac1*^*flox/flox*^; *Cre* mice, *Rac1*^*flox/flox*^ female mice were crossed with *Rac1*^+*/flox*^; *Cre* male mice. To obtain *Rac1*^*flox/flox*^; *Rac3*^−/−^, *Rac1*^*flox/flox*^; *Rac3*^+/−^; *Cre*, *Rac1*^+*/flox*^; *Rac3*^−/−^; *Cre* and *Rac1*^*flox/flox*^; *Rac3*^−/−^; *Cre* mice, *Rac1*^*flox/flox*^; *Rac3*^−/−^ female mice were crossed with *Rac1*^+*/flox*^; *Rac3*^+/−^; *Cre* male mice. Mice were genotyped by polymerase chain reaction (PCR) of genomic DNA extracted from tails using the following primers: 5′-TCCAATCTGTGCTGCCCATC-3′ and 5′-GATGCTTCTAGGGGTGAGCC-3′ for *Rac1*-floxed, 5′-CATTTCTGTGGCGTCGCCAAC-3′, 5′-CACGCGGCCGAGCTGTGGTG-3′ and 5′-TTGCTGGTGTCCAGACCAAT-3′ for *Rac3*-knockout, 5′-GCGGTCTGGCAGTAAAAACTATC-3′ and 5′-GTGAAACAGCATTGCTGTCACTT-3′ for GFAP-Cre. All animal experiments were performed in accordance with the relevant guidelines and regulations and approved by the Animal Care and Use Committee of Wakayama Medical University. ARRIVE guidelines were followed in all animal experiments.

### Histology

Cerebella were fixed in paraformaldehyde and embedded in paraffin. Sagittal and coronal sections were made with a microtome and subjected to Nissl staining, TUNEL staining and immunohistochemistry. The TUNEL method was carried out using Apoptag Fluorescein In Situ Apoptosis Detection Kit (Sigma-Aldrich, St. Louis, MO). For immunohistochemistry, the following primary antibodies were used in this study: rabbit anti-cleaved Caspase-3 (Cell Signaling Technology, Danvers, MA), rabbit anti-Ki67 (Abcam, Cambridge, UK), rabbit anti-NeuN (Cell Signaling Technology), mouse anti-phospho-histone H3 (Abcam), rabbit anti-GFAP (Proteintech, Rosemont, IL), mouse anti-BrdU (MBL, Tokyo, Japan), mouse anti-p27 (BD Transduction Laboratories, San Jose, CA), mouse anti-Sox2 (Proteintech), and mouse anti-Calbindin-D-28 K (Sigma-Aldrich). Immuno-positive signals were detected using Alexa Fluor 488 and 568-conjugated secondary antibodies (Thermo Fisher Scientific, Waltham, MA). Nuclei were visualized with DAPI, and sections were viewed and images were taken using an LSM700 confocal laser-scanning microscope (Zeiss, Oberkochen, Germany). Images of Nissl-stained sections were taken by a BZ9000 microscope (Keyence, Osaka, Japan).

### 5-Bromo-2′-deoxyuridine (BrdU) labeling

To assess the cell-cycle withdrawal, differentiation and migration of cerebellar granule neurons, mice were injected with 25 mg/kg of BrdU intraperitonially on postnatal day 7 (P7) and sacrificed 24, 48 and 72 h after the injection. Cerebella were fixed in paraformaldehyde and embedded in paraffin. Sagittal sections were subjected to immunohistochemistry mentioned above.

### Statistical analysis

For quantitation of the immunohistochemical results, 3 animals (2 sections/animal) were examined in each genotype. Immuno-positive signals were analyzed using ImageJ (https://imagej.nih.gov/ij/). Statistical analyses were carried out by one-way ANOVA followed by a Tukey’s post hoc test. Jarque–Bera test was used to determine whether sample data were normally distributed. Differences were defined as statistically significant when *P* < 0.05.

## Results

### Deletion of Rac in cerebellar granule neurons results in loss of granule neurons

**Figure 1:**
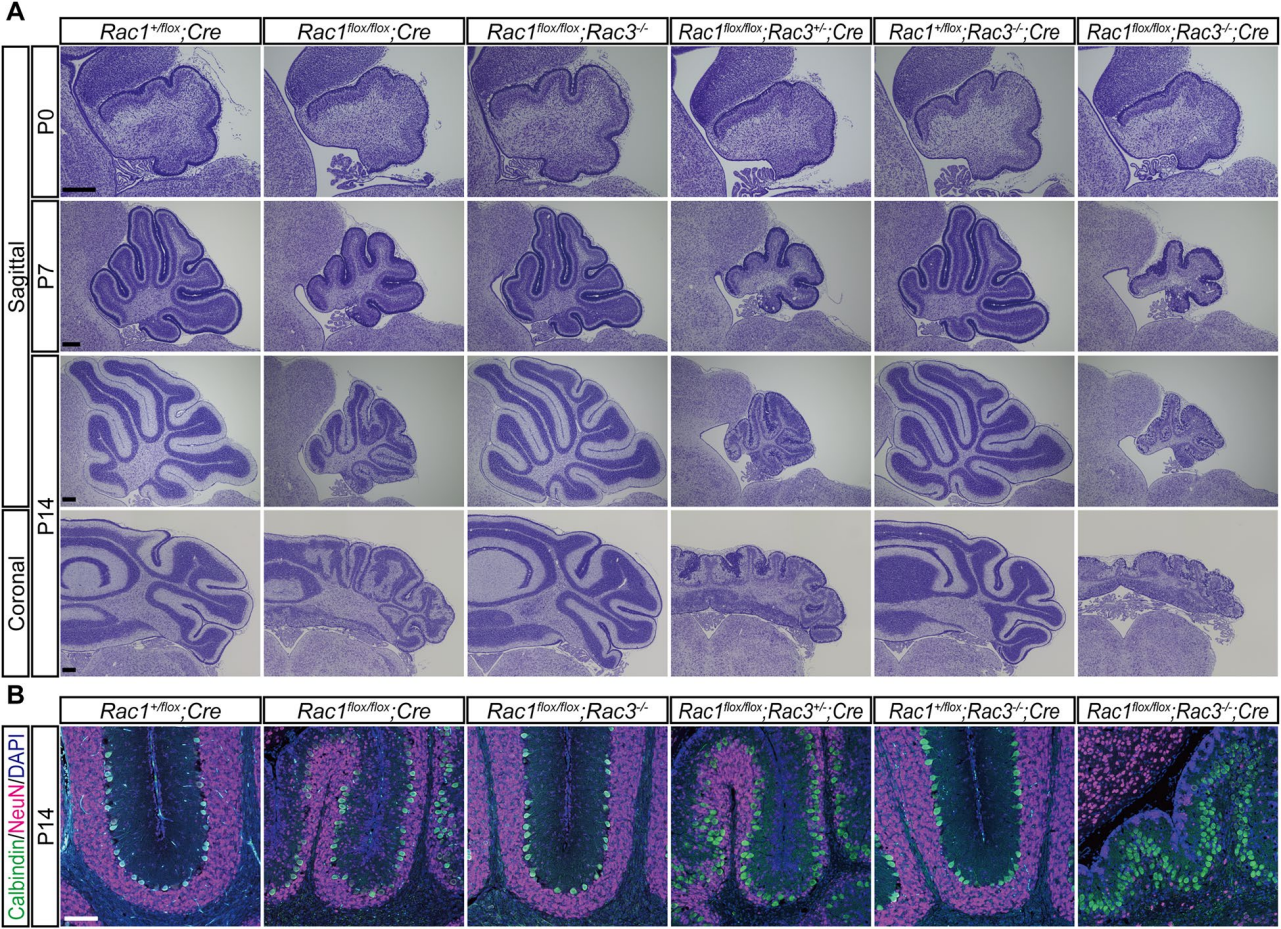
Deletion of Rac in cerebellar granule neurons results in loss of granule neurons. (**A**) Nissl-stained sections of the cerebellum on P0, P7 and P14. Development of the cerebellum was severely impaired in *Rac1*^*flox/flox*^; *Rac3*^−/−^; *Cre* mice. The cerebellum of *Rac1*^*flox/flox*^; *Rac3*^−/−^; *Cre* mice was slightly hypoplastic on P0, but it showed severe aplasia of the IGL in the entire cerebellum on P14. *Rac1*^*flox/flox*^; *Rac3*^+/−^; *Cre* mice showed intermediate phenotype between *Rac1*^*flox/flox*^; *Cre* and *Rac1*^*flox/flox*^; *Rac3*^−/−^; *Cre* mice. *Rac1*^+*/flox*^; *Rac3*^−/−^; *Cre* mice that retain only one allele of *Rac1* showed morphologically normal development of the cerebellum. (**B**) Immunohistochemistry for NeuN and Calbindin around preculminate fissure of the sagittal section. NeuN-positive granule neurons were almost completely absent in *Rac1*^*flox/flox*^; *Rac3*^−/−^; *Cre* mice on P14. n = 3 animals/genotype. Scale bars, 100 μm.

In this study, *Rac1* was deleted using GFAP-Cre mice, which induces Cre/loxP recombination in cerebellar granule neuron precursors from embryonic day 14.5 and also in Bergmann glial cells (Supplementary Fig. 1)^13–16^. At birth, cerebella of *Rac1*^*flox/flox*^; *Cre* mice were slightly smaller than those of *Rac1*^+*/flox*^; *Cre* mice (Fig. 1A). The difference in cerebellum size became larger as the mice grew up, and cerebella of *Rac1*^*flox/flox*^; *Cre* mice were markedly smaller than those of *Rac1*^+*/flox*^; *Cre* mice on P14. *Rac3*, a close homolog of *Rac1*, is expressed in the nervous system^4^. Although *Rac3*-knockout mice showed no obvious histological abnormalities in the brain^5^, *Rac3* may play some roles in the *Rac1*-deleted brain, *Rac1*/*Rac3* compound knockout (*Rac1*^*flox/flox*^; *Rac3*^−/−^; *Cre*) mice were therefore generated. The cerebella of *Rac1*^*flox/flox*^; *Rac3*^−/−^; *Cre* mice were much smaller than those of *Rac1*^*flox/flox*^; *Cre* mice on P14 (Fig. 1A). Sagittal and coronal sections revealed that not only anterior medial parts, but also caudal lateral parts of the cerebella were affected in *Rac1*^*flox/flox*^; *Rac3*^−/−^; *Cre* mice. Immunohistochemistry showed NeuN-positive cerebellar granule neurons were almost completely absent in *Rac1*^*flox/flox*^; *Rac3*^−/−^; *Cre* mice (Fig. 1B). The phenotype of *Rac1*^*flox/flox*^; *Rac3*^+/−^; *Cre* mice was milder than that of *Rac1*^*flox/flox*^; *Rac3*^−/−^; *Cre* mice (Fig. 1A, B). Presence of at least one allele of *Rac1* appeared sufficient to maintain cerebellum structures because overall morphology of the cerebellum of *Rac1*^+*/flox*^; *Rac3*^−/−^; *Cre* mice was normal (Fig. 1A, B). Rac is suggested by these results to be essential for the development of granule neurons in the entire cerebellum. Loss of *Rac3* itself has little effect on development of cerebellar granule neurons, but it can play some roles in *Rac1*-null situation. *Rac1*^*flox/flox*^; *Rac3*^−/−^; *Cre* mice were small and showed very severe ataxic gait on P14, so no further examinations were performed.

### Enhanced apoptotic cell death in the EGL of *Rac1*^*flox/flox*^; *Rac3*^+/−^; *Cre* and *Rac1*^*flox/flox*^; *Rac3*^−/−^; *Cre* mice

**Figure 2:**
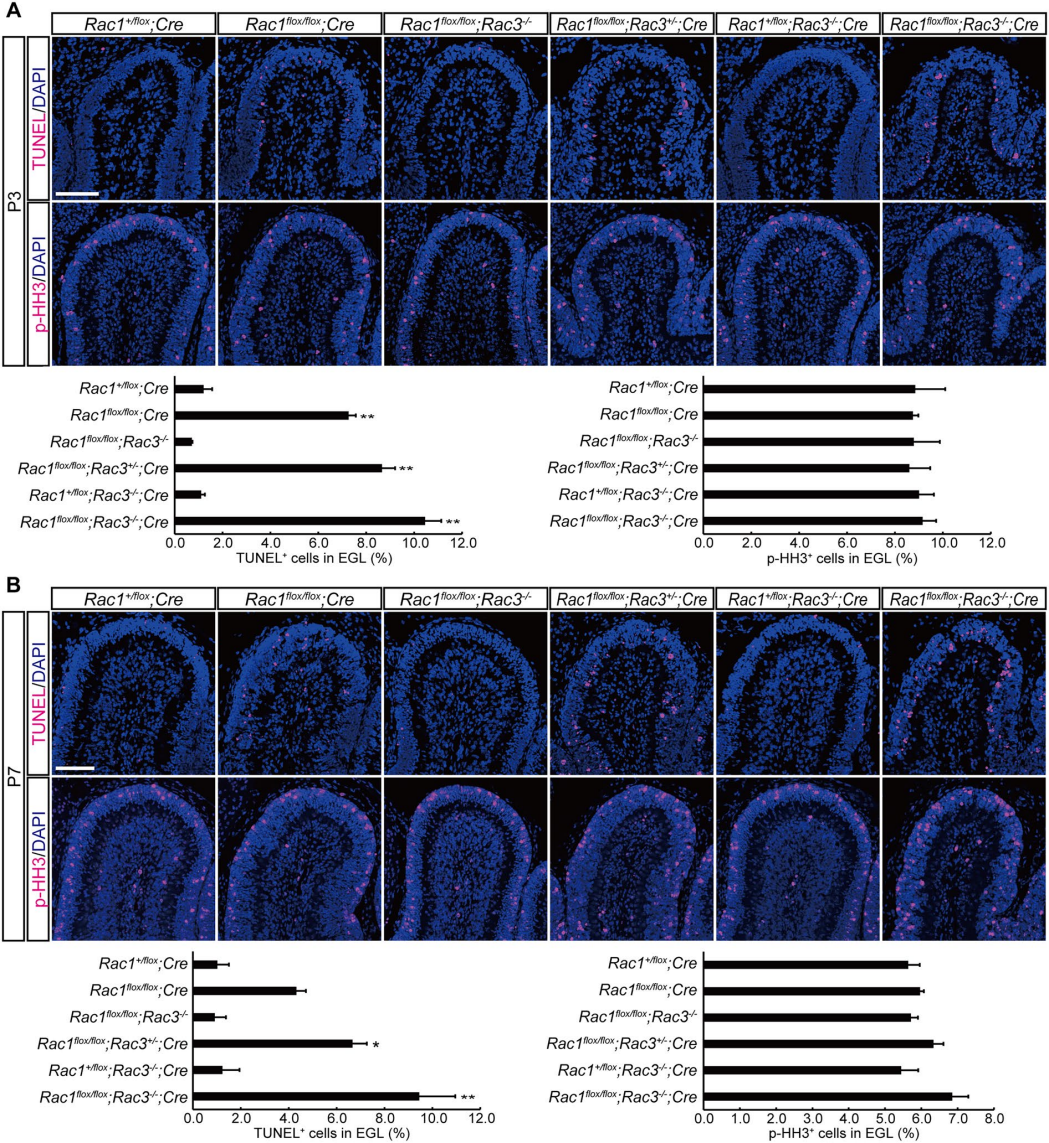
Enhanced apoptotic cell death in the EGL of *Rac1*^*flox/flox*^; *Rac3*^+/−^; *Cre* and *Rac1*^*flox/flox*^; *Rac3*^−/−^; *Cre* mice. TUNEL-staining and immunohistochemistry for phospho-histone H3 (p-HH3) of the sagittal section of the lobule IV/V on P3 (**A**) and P7 (**B**). The proportion of TUNEL-positive apoptotic cells was markedly increased in *Rac1*^*flox/flox*^; *Rac3*^+/−^; *Cre* and *Rac1*^*flox/flox*^; *Rac3*^−/−^; *Cre* mice, whereas that of p-HH3-positive mitotic cells was largely unaffected. Graphs depict the fraction of TUNEL-positive cells and p-HH3-positive cells in the EGL. Each bar represents mean + SD (n = 3). *P < 0.05, **P < 0.01 (significantly different from *Rac1*^+*/flox*^; *Cre* mice), one-way ANOVA followed by a Tukey’s post hoc test. Scale bars, 100 μm.

As granule neurons were lost in *Rac1*^*flox/flox*^; *Rac3*^−/−^; *Cre* mice, we next examined cell death and proliferation of granule neurons on P3 and P7. The proportion of TUNEL-positive apoptotic cells was markedly increased in the EGL of *Rac1*^*flox/flox*^; *Rac3*^+/−^; *Cre* and *Rac1*^*flox/flox*^; *Rac3*^−/−^; *Cre* mice on both P3 and P7 (Fig. 2A, B). TUNEL-positive cells in the cerebellum of *Rac1*^*flox/flox*^; *Rac3*^−/−^; *Cre* mice were observed in the inner most part of the EGL (Fig. 2A, B). Cell proliferation was assessed using immunohistochemistry for phospho-histone H3 (p-HH3) that labels M-phase cells, and the fraction of p-HH3-positive mitotic cells in the EGL was slightly increased in *Rac1*^*flox/flox*^; *Rac3*^+/−^; *Cre* and *Rac1*^*flox/flox*^; *Rac3*^−/−^; *Cre* mice on P7, but there were no statistically significant differences among genotypes (Fig. 2B). These results suggest that loss of NeuN-positive granule neurons in *Rac1*^*flox/flox*^; *Rac3*^−/−^; *Cre* mice is caused by enhanced apoptotic cell death in the EGL.

### Increased thickness of the EGL in *Rac1*^*flox/flox*^; *Cre*, *Rac1*^*flox/flox*^; *Rac3*^+/−^; *Cre* and *Rac1*^*flox/flox*^; *Rac3*^−/−^; *Cre* mice is caused by accumulation of p27 and NeuN-positive granule neurons

**Figure 3:**
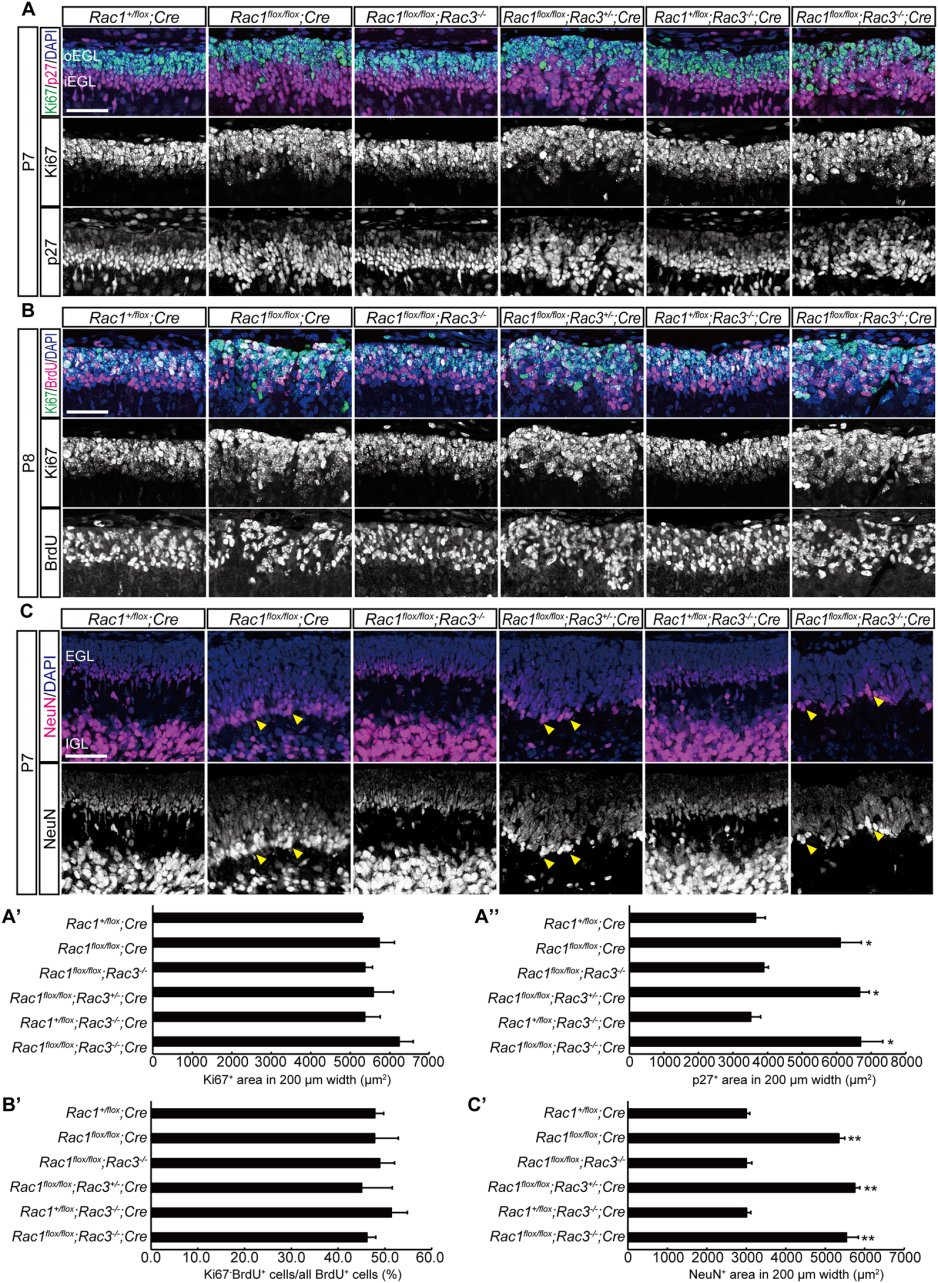
Thickness of the iEGL was increased in *Rac1*^*flox/flox*^; *Cre*, *Rac1*^*flox/flox*^; *Rac3*^+/−^; *Cre* and *Rac1*^*flox/flox*^; *Rac3*^−/−^; *Cre* mice due to impaired migration of NeuN-positive cells. (**A**) Immunohistochemistry for Ki67 and p27 of the sagittal section of the anterior part of the lobule IV on P7. (**B**) Immunohistochemistry for BrdU and Ki67 of the sagittal section of the anterior part of the lobule IV. Mice were injected with BrdU on P7 and sacrificed 24 h after the injection (P8). (**C**) Immunohistochemistry for NeuN of the sagittal section of the anterior part of the lobule IV on P7. Immunopositive signal of NeuN was strong in granule neurons in the IGL but weak in those in the EGL in *Rac1*^+*/flox*^; *Cre*, *Rac1*^*flox/flox*^; *Rac3*^−/−^ and *Rac1*^+*/flox*^; *Rac3*^−/−^; *Cre* mice. Some NeuN-positive cells in the EGL showed strong immunoreactivity in *Rac1*^*flox/flox*^; *Cre*, *Rac1*^*flox/flox*^; *Rac3*^+/−^; *Cre* and *Rac1*^*flox/flox*^; *Rac3*^−/−^; *Cre* mice (arrowheads). (**A**′ and **A**″) The oEGL area occupied by Ki67-positive cells was not different among genotypes, whereas the iEGL area occupied by p27-positive cells was significantly increased in *Rac1*^*flox/flox*^*Cre*, *Rac1*^*flox/flox*^; *Rac3*^+/−^; *Cre* and *Rac1*^*flox/flox*^; *Rac3*^−/−^; *Cre* mice. (**B**′) Cell-cycle exit was not affected by Rac depletion/deletion. The proportion of cells that exit the cell cycle (BrdU^+^/Ki67^-^) among all BrdU^+^ cells was not changed among genotypes. (**C**′) The area occupied by NeuN-positive cells was significantly increased in *Rac1*^*flox/flox*^*Cre*, *Rac1*^*flox/flox*^; *Rac3*^+/−^; *Cre* and *Rac1*^*flox/flox*^; *Rac3*^−/−^; *Cre* mice. Each bar represents mean + SD (n = 3). *P < 0.05, **P < 0.01 (significantly different from *Rac1*^+*/flox*^; *Cre* mice), one-way ANOVA followed by a Tukey’s post hoc test. Scale bars, 50 μm.

In spite of enhanced cell death, the EGL of *Rac1*^*flox/flox*^; *Cre*, *Rac1*^*flox/flox*^; *Rac3*^+/−^; *Cre* and *Rac1*^*flox/flox*^; *Rac3*^−/−^; *Cre* mice appeared thicker than that of the other genotypes on P7 (Fig. 2B), so cellular components of the EGL were examined next. The EGL is divided into an outer layer (oEGL) of proliferating progenitors and inner layer (iEGL) of postmitotic neurons^17^. We examined arrangement of oEGL and iEGL by immunohistochemistry for Ki67 (oEGL cell marker) and p27 (iEGL cell marker). The iEGL area occupied by p27-positive cells was significantly increased in *Rac1*^*flox/flox*^; *Cre*, *Rac1*^*flox/flox*^; *Rac3*^+/−^; *Cre* and *Rac1*^*flox/flox*^; *Rac3*^−/−^; *Cre* mice, whereas the oEGL area occupied by Ki67-positive cells was not different among genotypes (Fig. 3A, A′, A″). In *Rac1*^+*/flox*^; *Cre*, *Rac1*^*flox/flox*^; *Rac3*^−/−^ and *Rac1*^+*/flox*^; *Rac3*^−/−^; *Cre* mice, Ki67-positive cells and p27-positive cells were clearly separated into outer and inner compartments, respectively. However, these two cell populations were intermingled in *Rac1*^*flox/flox*^; *Cre*, *Rac1*^*flox/flox*^; *Rac3*^+/−^; *Cre* and *Rac1*^*flox/flox*^; *Rac3*^−/−^; *Cre* mice (Fig. 3A). To examine whether accumulation of p27-positive cells is caused by the accelerated differentiation of progenitor cells, cell-cycle withdrawal of progenitor cells was evaluated. BrdU was injected on P7 and the proportion of cells that exit the cell cycle after 24 h (P8) was counted, which was calculated as the ratio of BrdU^+^/Ki67^−^ cells among all BrdU-labeled cells. Cell-cycle exit and differentiation into postmitotic neurons of precursors was not altered in *Rac1*^*flox/flox*^; *Cre*, *Rac1*^*flox/flox*^; *Rac3*^+/−^; *Cre* and *Rac1*^*flox/flox*^; *Rac3*^−/−^; *Cre* mice (Fig. 3B, B′). Accumulation of p27-positive cells in the EGL is suggested by these results not to be caused by the enhanced differentiation of precursor cells to postmitotic neurons. The inner most part of the EGL, p27-positive cells begin to express NeuN and migrate into the molecular layer to form the IGL. The accumulation of p27-positive cells in the EGL may be caused by impaired migration of NeuN-positive cells, so NeuN expression in the EGL was examined next. The NeuN-positive area in the EGL was significantly increased in *Rac1*^*flox/flox*^; *Cre*, *Rac1*^*flox/flox*^; *Rac3*^+/−^; *Cre* and *Rac1*^*flox/flox*^; *Rac3*^−/−^; *Cre* mice (Fig. 3C, C′). Rac-depleted/deleted granule neurons are suggested to differentiate normally as far as they express NeuN but exhibit impaired migration into the molecular layer, resulting in accumulation of p27 and NeuN-positive cells in the EGL. Immunopositive signal of NeuN was strong in granule neurons in the IGL but relatively weak in those in the EGL in *Rac1*^+*/flox*^; *Cre*, *Rac1*^*flox/flox*^; *Rac3*^−/−^ and *Rac1*^+*/flox*^; *Rac3*^−/−^; *Cre* mice. Conversely, some NeuN-positive cells in the EGL showed strong immunoreactivity in *Rac1*^*flox/flox*^; *Cre*, *Rac1*^*flox/flox*^; *Rac3*^+/−^; *Cre* and *Rac1*^*flox/flox*^; *Rac3*^−/−^; *Cre* mice (Fig. 3C, arrowheads). NeuN-positive cells that cannot migrate into the molecular layer may therefore differentiate into cells similar to granule neurons in the IGL in the EGL.

### Rac-deficient granule neurons die before they migrate to the IGL

**Figure 4:**
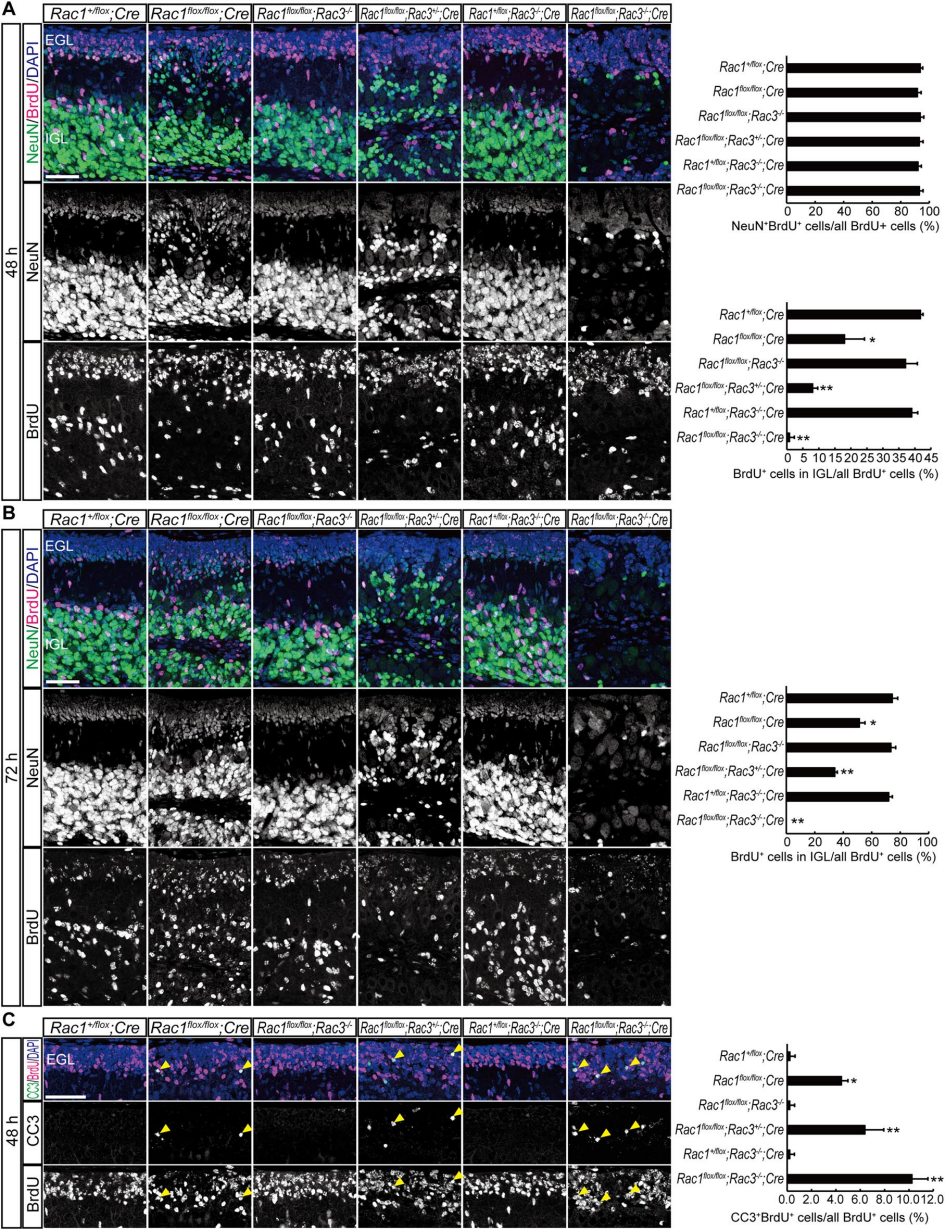
Newly generated granule neurons can differentiate into NeuN-positive cells but hardly reach to the IGL in *Rac1*^*flox/flox*^; *Rac3*^−/−^; *Cre* mice. Immunohistochemistry for BrdU and NeuN of the sagittal section of the anterior part of the lobule IV (**A**, **B**). Mice were injected with BrdU on P7 and sacrificed 48 h (**A**) or 72 h (**B**) after the injection. Most of the BrdU-incorporated cells differentiate into NeuN-positive cells in 48 h in each genotype. Significantly fewer BrdU-incorporated cells migrate to the IGL in *Rac1*^*flox/flox*^; *Cre*, *Rac1*^*flox/flox*^; *Rac3*^+/−^; *Cre* and *Rac1*^*flox/flox*^; *Rac3*^−/−^; *Cre* mice at 48 h and 72 h. Immunohistochemistry for BrdU and cleaved Caspase-3 (CC3) of the sagittal section of the anterior part of the lobule IV (**C**). Mice were injected with BrdU on P7 and sacrificed 48 h after the injection. The proportion of CC3^+^/BrdU^+^ cells (arrowheads) among all BrdU^+^ cells was significantly increased in *Rac1*^*flox/flox*^; *Cre*, *Rac1*^*flox/flox*^; *Rac3*^+/−^; *Cre* and *Rac1*^*flox/flox*^; *Rac3*^−/−^; *Cre* mice. Each bar represents mean + SD (n = 3). *P < 0.05, **P < 0.01 (significantly different from *Rac1*^+*/flox*^; *Cre* mice), one-way ANOVA followed by a Tukey’s post hoc test. Scale bars, 50 μm.

To confirm whether Rac-deficient granule neurons differentiate as far as NeuN expression and die in the inner most part of the EGL, mice were injected with BrdU on P7 and differentiation and migration of BrdU-incorporated granule neurons were examined. A large volume of the BrdU-incorporated granule neurons differentiated to NeuN-positive cells in 48 h in all the genotypes (Fig. 4A). However, significantly fewer BrdU-incorporated cells migrated to the IGL (past the Purkinje cell layer) in *Rac1*^*flox/flox*^; *Cre*, *Rac1*^*flox/flox*^; *Rac3*^+/−^; *Cre* and *Rac1*^*flox/flox*^; *Rac3*^−/−^; *Cre* mice compared with other genotypes at 48 h (Fig. 4A) and 72 h (Fig. 4B). Only a few BrdU-incorporated cells survived in *Rac1*^*flox/flox*^; *Rac3*^−/−^; *Cre* mice at 72 h (Fig. 4B). In addition, some BrdU-positive cells in the EGL of *Rac1*^*flox/flox*^; *Cre*, *Rac1*^*flox/flox*^; *Rac3*^+/−^; *Cre* and *Rac1*^*flox/flox*^; *Rac3*^−/−^; *Cre* mice were also positive for cleaved Caspase-3 (CC3), an apoptotic cell marker, at 48 h after the BrdU injection (Fig. 4C, arrowheads). The proportion of CC3^+^/BrdU^+^ cells among all BrdU^+^ cells was significantly increased in these mice. Rac-deficient granule neurons can therefore differentiate almost normally as far as NeuN expression but they die before they migrate to the IGL. Migration of granule neurons was impaired in *Rac1*^*flox/flox*^; *Cre* and *Rac1*^*flox/flox*^; *Rac3*^+/−^; *Cre* mice, and the phenotype was much more severe in *Rac1*^*flox/flox*^; *Rac3*^+/−^; *Cre* mice than that in *Rac1*^*flox/flox*^; *Cre* mice, suggesting that Rac is required for the migration of granule neurons and that *Rac3* has some roles in *Rac1*-deficient granule neurons in terms of migration. Existence of NeuN-positive cells between the EGL and IGL in *Rac1*^*flox/flox*^; *Cre* and *Rac1*^*flox/flox*^; *Rac3*^+/−^; *Cre* mice also suggests impaired migration of granule neurons in these mice (Fig. 4A, B).

### Loss of Rac caused disorganization of processes of Bergmann Glia

**Figure 5:**
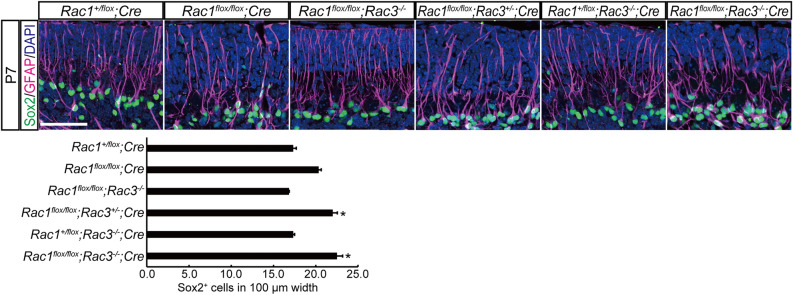
Misalignment of Bergmann glial processes in *Rac1*^*flox/flox*^; *Cre*, *Rac1*^*flox/flox*^; *Rac3*^+/−^; *Cre* and *Rac1*^*flox/flox*^; *Rac3*^−/−^; *Cre* mice. Immunohistochemistry for GFAP and Sox2 of the sagittal section of the anterior part of the lobule IV on P7. GFAP-positive processes of Bergman glial cells appeared disorganized in *Rac1*^*flox/flox*^; *Cre*, *Rac1*^*flox/flox*^; *Rac3*^+/−^; *Cre* and *Rac1*^*flox/flox*^; *Rac3*^−/−^; *Cre* mice. Sox2-positive cells in 100 μm width were slightly increased in *Rac1*^*flox/flox*^; *Rac3*^+/−^; *Cre* and *Rac1*^*flox/flox*^; *Rac3*^−/−^; *Cre* mice. Each bar represents mean + SD (n = 3). *P < 0.05, (significantly different from *Rac1*^+*/flox*^; *Cre* mice), one-way ANOVA followed by a Tukey’s post hoc test. Scale bar, 50 μm.

The GFAP-Cre mouse line used in this study induces Cre/loxP recombination not only in granule neurons, but also in Bergmann glial cells^15,16^. Several findings suggest that Bergmann glia works as a scaffold for migrating granule neurons^18^. The arrangement of processes of Bergmann glia was therefore examined by immunostaining for GFAP. GFAP-positive processes of Bergmann glial cells in *Rac1*^*flox/flox*^; *Cre, Rac1*^*flox/flox*^; *Rac3*^+/−^; *Cre* and *Rac1*^*flox/flox*^; *Rac3*^−/−^; *Cre* mice appeared disorganized probably due to hypoplasia/aplasia of the IGL (Fig. 5). We also immunostained Sox2, which labels nuclei of Bergmann glial cells^19,20^, and counted the number of Sox2-positive cells. The number of Sox2-positive cells in 100 μm width was significantly increased in *Rac1*^*flox/flox*^; *Rac3*^+/−^; *Cre* and *Rac1*^*flox/flox*^; *Rac3*^−/−^; *Cre* mice. Unlike granule neurons, loss of Rac barely affected the survival of Bergman glial cells. The density of Bergmann glial cells was increased, probably due to the hypoplasia of the cerebellar lobules in *Rac1*^*flox/flox*^; *Rac3*^+/−^; *Cre* and *Rac1*^*flox/flox*^; *Rac3*^−/−^; *Cre* mice. Loss of Rac1 in Bergmann glia reportedly had subtle effect on the organization of glial fibers and migration of granule neurons^7,21^. Furthermore, Rac3 is absent in glial lineage cells including Bergmann glia^8,21^, it is unlikely that loss of Rac in Bergmann glia primarily contributes to the impaired migration and loss of granule neurons in *Rac1*^*flox/flox*^; *Rac3*^−/−^; *Cre* mice. However, the possibility that misalignment of Bergmann glial fibers caused by hypoplasia/aplasia of the IGL secondarily contributes to the impaired migration and loss of granule neurons cannot be ruled out.

## Discussion

Deletion of both *Rac1* and *Rac3* was shown to be almost completely absent of granule neurons in the cerebellum. Rac-deficient granule neurons differentiate as far as NeuN expression but die in the inner most part of the EGL and cannot migrate to the IGL. Loss of *Rac3* itself has little effect on the development of cerebellar granule neurons, but *Rac1*^*flox/flox*^; *Rac3*^−/−^; *Cre* mice showed much more severe phenotype than that of *Rac1*^*flox/flox*^; *Cre* mice. In addition, *Rac1*^*flox/flox*^; *Rac3*^+/−^; *Cre* mice showed intermediate phenotype between *Rac1*^*flox/flox*^; *Cre* and *Rac1*^*flox/flox*^; *Rac3*^−/−^; *Cre* mice. Rac is suggested by our results to be required for both survival and migration of granule neurons and that *Rac3* likely plays roles in *Rac1*-deleted neurons. Meanwhile, overall morphology of the cerebellum of *Rac1*^+*/flox*^; *Rac3*^−/−^; *Cre* mice was normal. Presence of at least one allele of *Rac1* is therefore sufficient to promote development of cerebellar granule neurons. In the inner-most part of the EGL, granule neurons begin to express NeuN and migrate into the molecular layer to form the IGL. The immunoreactivity of NeuN is weaker in granule neurons in the EGL than those in the IGL. However, some NeuN-positive granule neurons in the EGL of *Rac1*^*flox/flox*^; *Rac3*^−/−^; *Cre* mice showed strong immunoreactivity, similar to those in the IGL in other genotypes. Rac-deficient granule neurons that cannot migrate into the molecular layer can differentiate into mature cells in terms of marker expression. However, it is difficult to determine whether granule neurons die because they cannot make complete differentiation or because they cannot migrate to the IGL and are mis-localized. The precise cause of the cell death of Rac-deficient granule neurons remains unknown.

Small Rho GTPases including Rac are known to have important roles in the development of cerebellar granule neurons both in cell autonomously and non-cell autonomously. For example, deletion of RhoA or Cdc42 in granule neurons delayed their migration^17,22^. In Bergman glia, deletion of Cdc42, but not Rac1, impaired migration of granule neurons, but compound deletion of both Cdc42 and Rac1 much more severely impaired the migration of granule neurons than that of Cdc42-deletion alone^21^. Furthermore, in granule neurons, deletion of β-chimaerin, a GTPase activating protein (GAP) of Rac, or Trio, a guanine nucleotide exchanging factor (GEF) of RhoA, RhoG and Rac, caused defective migration^23,24^. Disruption of Abr and Bcr, RacGAPs, caused misalignment of Bergmann processes and granule cell migration defects^25^. These findings together with ours suggest that small Rho GTPases not only in granule neurons themselves but also in Bergman glial cells play very important roles in the development of cerebellar granule neurons, especially in their migration. The activity of each small Rho GTPase must be tightly regulated.

Deletion of both *Rac1* and *Rac3* in the cerebellum was also previously shown to cause severely impaired development of granule neurons^8^. The biggest difference between current and previous studies is the distribution of hypoplasia/aplasia in the cerebellum. In *Atoh1-Cre*; *Rac1*^*flox/flox*^; *Rac3*^*−/−*^ mice, agenesis of the IGL was observed only in the anterior medial part of the cerebellum^8^, but in our *Rac1*^*flox/flox*^; *Rac3*^−/−^; *GFAP-Cre* mice, it was observed in the entire cerebellum. Furthermore, degeneration of cerebellar granule neurons was much more severe in *Rac1*^*flox/flox*^; *Rac3*^−/−^; *GFAP-Cre* mice than in *Atoh1-Cre*; *Rac1*^*flox/flox*^; *Rac3*^*−/−*^ mice. Deletion of receptor for activated C kinase (Rack1) by GFAP-Cre driver resulted in much more severe phenotype than that by Atoh1-Cre driver^16^. Rack1 deletion by GFAP-Cre mice resulted in agenesis of lobules throughout the rostral to caudal parts of the medial vermis. On the other hand, loss of Rack1 by Atoh1-Cre mice affected only the rostral part of the cerebellum. This difference may be caused by the difference in Cre recombinase activity in GFAP-Cre and Atoh1-Cre mice. Cre recombinase activity in the cerebellum of Atoh1-Cre mice is higher in the rostral part compared with in the caudal regions^16^. By deletion of *Rac1* in the entire cerebellum by GFAP-Cre driver, Rac was shown in our study to play crucial roles in the development of cerebellar granule neurons not only in the anterior medial part but in the entire cerebellum. Nakamura et al.^8^ identified *Mid1* as a downstream target of Rac to promote cerebellar development. The human ortholog is a responsible gene for Opitz G/BBB syndrome characterized by malformation of midline structures^26^. In addition, *Mid1*-deficient mice showed hypoplasia of the cerebellum in the anterior medal portion only^27^. Other downstream targets of Rac may play an important role in the caudal lateral part of the cerebellum.

Deletion of *Rac1* in telencephalic neuroepithelium resulted in accelerated cell cycle exit of progenitors^28^. Conversely, deletion of *Rac1* or *Rac1*/*Rac3* in the medial ganglionic eminence reduced cell cycle exit of interneuron progenitors^29,30^. Nakamura et al.^8^ and our group both showed no effect of deletion of both *Rac1* and *Rac3* in the cerebellum on cell cycle exit of granule neuron progenitors. Rac may play different roles in cell cycle exit of progenitors depending on cell types.

Rac plays a variety of roles during development of the nervous system, including neurogenesis, differentiation, migration, dendritogenesis, axon guidance and synapse formation^1,2^, but there is poor current understanding of downstream signaling pathways being involved in each process. In the central nervous system, *Rac3*, a close homolog of *Rac1*, is ubiquitously expressed, and it may have roles in *Rac1*-deficient neurons, making it difficult to recognize the functions of “Rac” and its downstream targets. The present study showed that deletion of both *Rac1* and *Rac3* in cerebellar granule neurons resulted in much more severe impairment in survival and migration than that of *Rac1*-deletion alone. Our experimental system can therefore be considered to be an effective tool for identification of downstream targets of Rac in neuronal survival and migration.

## Supplementary Information

Supplementary Figure 1.

Supplementary Figure 2.

Supplementary Legends.

## Data Availability

The data that support the findings of this study are available from the corresponding author upon reasonable request.
