# Supplementary figures and images for: Rac-deficient cerebellar granule neurons die before they migrate to the internal granule layer
Source: Sci Rep. 2022 Sep 1;12:14848. doi: 10.1038/s41598-022-19252-y (PMC9436960; doi:10.1038/s41598-022-19252-y)

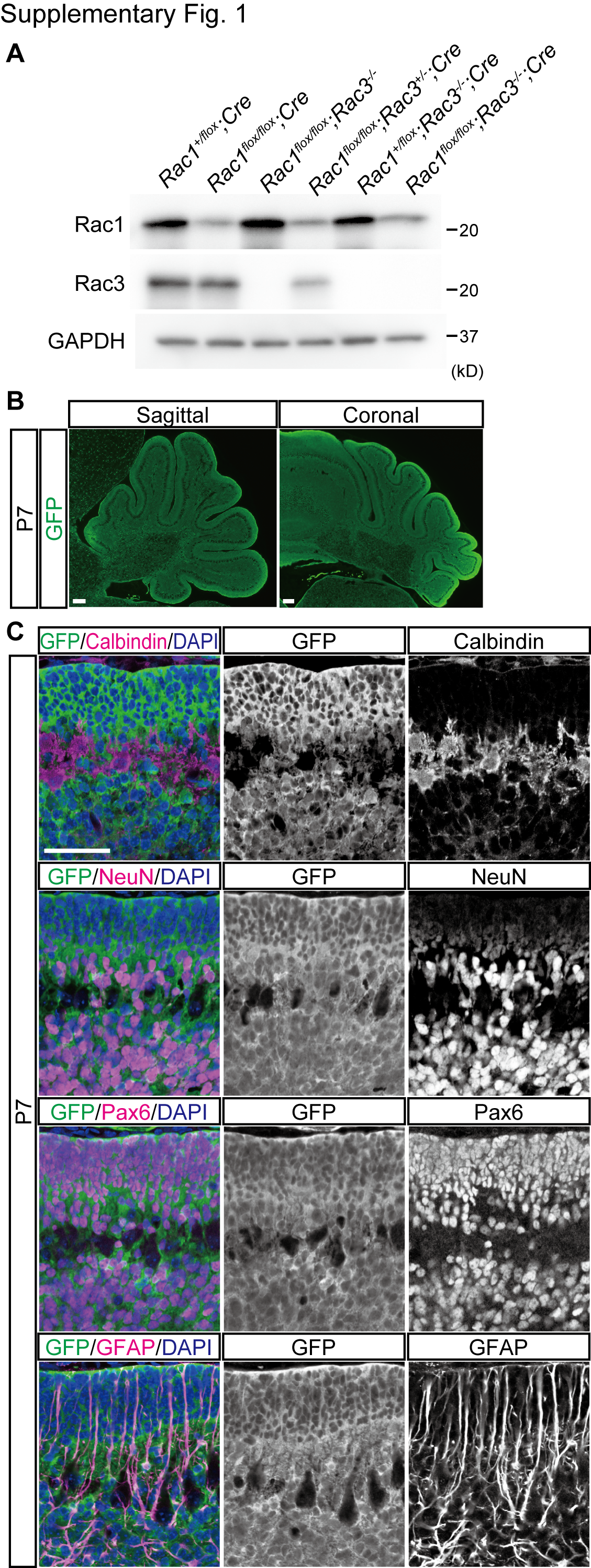

Supplement: Supplementary file 1 — Supplementary Figure 1. [file 41598_2022_19252_MOESM1_ESM.tif]

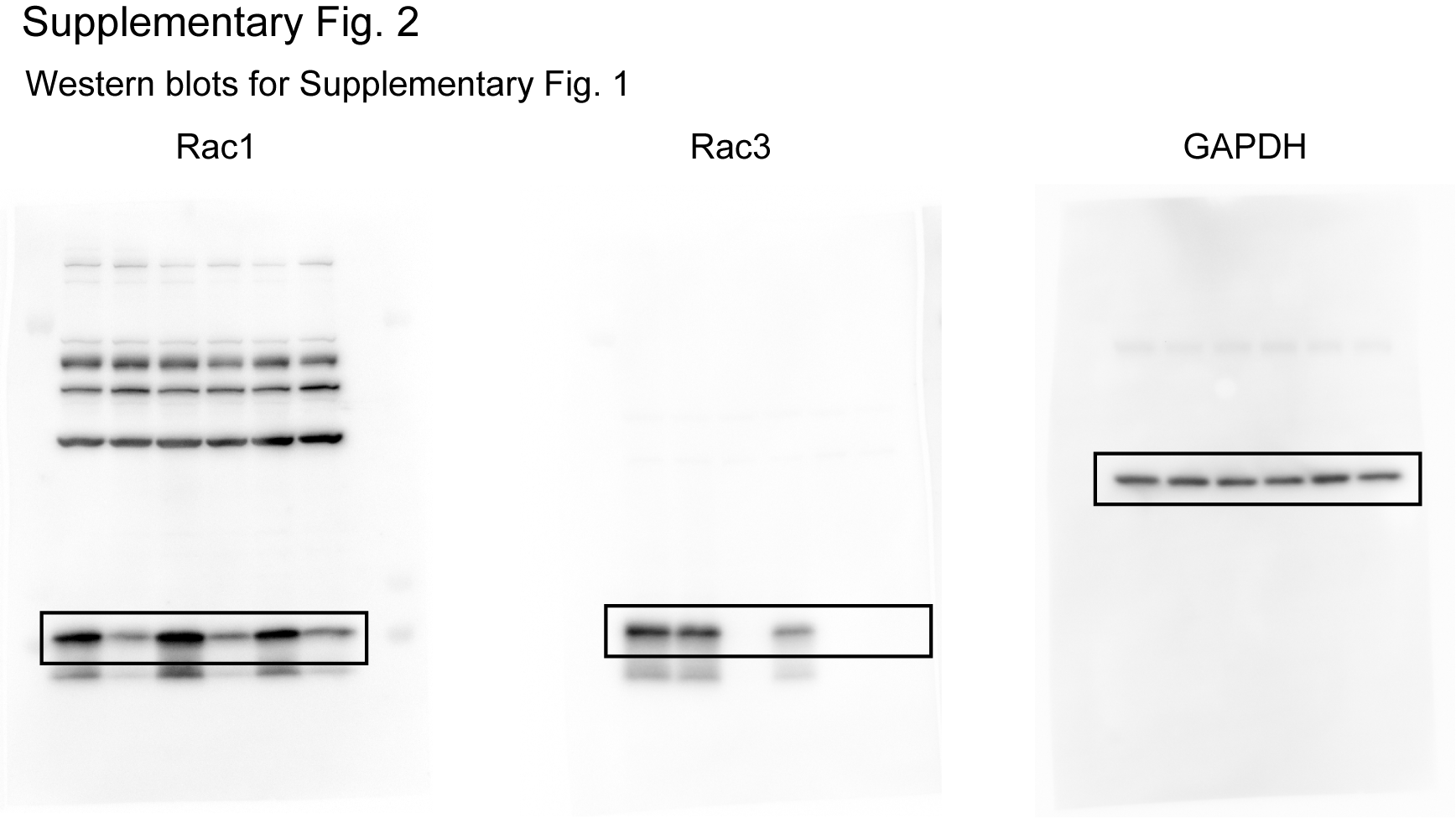

Supplement: Supplementary file 2 — Supplementary Figure 2. [file 41598_2022_19252_MOESM2_ESM.tif]
