# Supplementary material for: Rac-deficient cerebellar granule neurons die before they migrate to the internal granule layer
Source: Sci Rep. 2022 Sep 1;12:14848. doi: 10.1038/s41598-022-19252-y (PMC9436960; doi:10.1038/s41598-022-19252-y)
Supplement: Supplementary file 3 — Supplementary Legends. [file 41598_2022_19252_MOESM3_ESM.docx]

**Supplementary Figure legends**

Supplementary Fig. 1. Deletion of Rac1 by GFAP-Cre driver. (A) Deletion of Rac1 and Rac3 was confirmed by Western blot of protein extract from P7 cerebella. (B) GFAP-Cre dependent Cre/loxP-recombined cells were visualized by crossing GFAP-Cre mice with STOP-floxed EYFP reporter (R26R-EYFP) mice. EYFP-positive signals were detected by immunohistochemistry using anti-GFP antibody. GFP-positive signals were observed in the entire cerebellum on P7. (C) GFP-positive signals were overlapped with those of NeuN (granule neuron), Pax6 (granule neuron precursor), and GFAP (Bergmann glia) but did not colocalized with those of Calbindin (Purkinje cell). Scale bars, 100 μm.
